# Adaptive-Cognitive Kalman Filter and Neural Network for an Upgraded Nondispersive Thermopile Device to Detect and Analyze Fusarium Spores

**DOI:** 10.3390/s19224900

**Published:** 2019-11-09

**Authors:** Son Pham, Anh Dinh

**Affiliations:** Department of Electrical and Computer Engineering, University of Saskatchewan, Saskatoon, SK S7N 5A9, Canada; son.pham@usask.ca

**Keywords:** burst noise, outlier, thermal noise, Kalman, filter, neural network, thermopile, Fusarium detection

## Abstract

Noises such as thermal noise, background noise or burst noise can reduce the reliability and confidence of measurement devices. In this work, a recursive and adaptive Kalman filter is proposed to detect and process burst noise or outliers and thermal noise, which are popular in electrical and electronic devices. The Kalman filter and neural network are used to preprocess data of three detectors of a nondispersive thermopile device, which is used to detect and quantify Fusarium spores. The detectors are broadband (1 µm to 20 µm), $\lambda_{1}$ (6.09 ± 0.06 µm) and $\lambda_{2}$ (9.49 ± 0.44 µm) thermopiles. Additionally, an artificial neural network (NN) is applied to process background noise effects. The adaptive and cognitive Kalman Filter helps to improve the training time of the neural network and the absolute error of the thermopile data. Without applying the Kalman filter for $\lambda_{1}$ thermopile, it took 12 min 09 s to train the NN and reach the absolute error of 2.7453 × 10^4^ (n. u.). With the Kalman filter, it took 46 s to train the NN to reach the absolute error of 1.4374 × 10^4^ (n. u.) for $\lambda_{1}$ thermopile. Similarly, to the $\lambda_{2}$ (9.49 ± 0.44 µm) thermopile, the training improved from 9 min 13 s to 1 min and the absolute error of 2.3999 × 10^5^ (n. u.) to the absolute error of 1.76485 × 10^5^ (n. u.) respectively. The three-thermopile system has proven that it can improve the reliability in detection of Fusarium spores by adding the broadband thermopile. The method developed in this work can be employed for devices that encounter similar noise problems.

## 1. Introduction

*Fusarium* is a hazardous fungus. It can weaken the immunization system of the hosts such as animals and human. It also and cause different diseases such as onychomycosis or keratitis for human [1], or meningoencephalitis in the dog [2]. *Fusarium* also can result in many other diseases on plants such as *Fusarium* wilt on watermelon or bean [3,4], *Fusarium* head blight on wheat [5], *Fusarium* dry on citrus [6] or *Fusarium* root rot [7]. According to *Fusarium* management guide [8], *Fusarium* head blight disease, which is the key factor to cause *Fusarium* damage kernel on wheat, has annually resulted in losses of hundreds of million dollars. Many other previous studies of analyzing and detection *Fusarium* were conducted by applying mass spectroscopy [9], Fourier transform infrared spectroscopy, near-infrared spectroscopy [10,11], polymerase-chain-reaction machine [12], chlorophyll fluorescent imaging [5] or impedance-based gold-electrodes sensor [13]. Though these mentioned approaches are effective, some drawbacks can be seen such as expensive, complex to manipulate and hard to achieve quick detection. Thus, early detecting of *Fusarium* spore help crops to avoid dangerous fungal diseases and losses. *Fusarium* spores can spread out through the water, air and collaborative media of both water and air [14,15,16]. Based on dispersal mechanisms, it can be sorted in a one-phase mechanism or two-phase mechanism. The one-phase mechanism means that spores can be dispersed by merely air or water. The two-phase mechanism means that the spores can be dispersed by the cooperation of air and water in the form of water drops in wind or bubbles in raining water containing spores [14,15,16,17,18,19].

From the studies pertaining to *Fusarium*, *Fusarium* spores can be dispersed most through the air phase. From this feature, the *Fusarium* detection method and device proposed in [20] were suggested and designed. In this research, based on the Beer–Lambert law [21], the group-distinction coefficient (GDC) was proposed to distinguish the substances. The group-distinction coefficient was calculated by using signals from two infrared narrow-bandwidth thermopiles. The detection method and the device were proved that worked well. However, the authors encountered some difficulties which are similar GDC values and system noises. In the research in [20], the studied samples were *Fusarium oxysporum* chlamydospores [22], pollen, starch and turmeric, in which, the GDCs of *Fusarium* and starch were very close to each other. The noises are background noise, thermal noise and burst noise [20].

There are many different types of noise such as thermal, background, burst, flicker and avalanche noises [23,24]. These noises mainly occur in electronic or electrical devices and can be processed and treated in different ways to reduce the effects caused by them to the performance of devices. A thermal noise or Johnson noise, which is thermal agitation of electrons within electronic components can be reduced by an analog or digital filter [24,25,26]. Background (BG) noise or direct current (DC) noise can occur in amplifier circuits as they need bias currents to work, and the currents can be changed by the operating conditions such as temperature [27]. Additionally, BG noise can be caused by input offset voltage along with bias currents of the operational amplifiers [9]. Burst or popcorn noise can happen in semiconductors and is unpredictable [24]. This type of noise can cause outliers in data, as a result, outlier detection and treatment are crucial tasks. In the study on outlier detection [28], Dan L. et al., presented a Haar wavelet transform method to detect burst noise based on the singularity of the noise. In a different work, to detect outliers that were from data in the frequency domain, Deschrijver, D. et al., [29] suggested a modified vector fitting algorithm by solving the least-squares equations of a set of scattering parameter data samples.

This paper proposes a novel method to detect *Fusarium*, to distinguish two substances with similar GDCs, and to introduce techniques to reduce thermal and burst noises or outliers in the data collected from the thermopiles. The method in this paper is upgraded from the previous work. To process both thermal and burst noise, an adaptive and cognitive Kalman filter (ACKF) is proposed. In the filter, a mechanism of outlier detection indicates the outlier positions and the filter will eliminate the outliers. As BG noise affects the impulsive signals or peak data (PD), the PD should be processed to eliminate the effect of the BG noise. The PD with the noise or error are eliminated by an artificial neural network (NN). From [20], the two narrow-bandwidth thermopiles, $\lambda_{1}$ (6.09 ± 0.06 µm) and $\lambda_{2}$ (9.49 ± 0.44 µm), were used. In this research, the third thermopile was added. This add-on thermopile is a broadband (BR) spectrum detector (1 µm to 20 µm), which was upgraded from a reference sensor of monitoring the IR light source [20]. The thermopiles BR, $\lambda_{1}$, and $\lambda_{2}$ can be used to analyze samples. The rest of the paper is structured as follows. Section 2 is the background of the Kalman algorithm and the neural network. Section 3 is about the outlier detection and adaptation mechanism for the Kalman filter. Additionally, it also discusses the NN approach. Section 4 provides the results and discussion. Lastly, Section 5 concludes the work.

## 2. Background of the Applied Algorithms

### 2.1. Kalman Algorithm

**Figure 1:**
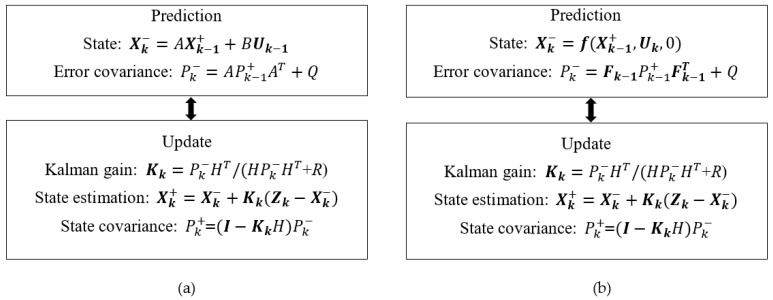
Kalman algorithm operation diagram. (**a**): Kalman and (**b**): extended Kalman.

Kalman algorithm is a versatile tool as it can be applied in many applications such as tracking objects (body parts, missiles, etc.) [30,31,32,33], navigation [34], error data correction [35] or finance [36]. Kalman algorithm always has two distinct stages: prediction and measurement. Kalman is an optimal algorithm, as it can continuously improve the system outputs based on a recursive method of calculating the error covariance and prediction. With a linear system in the state-space model, the discrete Kalman can be applied. The discrete-time state evolution equation of a linear system [37] can be defined as:

(1) $$X_{k}=AX_{k-1}+BU_{k}+W_{k},$$

where *A* is the state transition matrix impacting on $X_{k-1}$, which is the state vector at the discrete-time ***k*** − 1; *B* is the control-input matrix; $U_{k}$ and is the control vector and $W_{k}$ is the process noise vector, which is supposed to be zero-mean Gaussian with the process noise covariance matrix *Q*, $W_{k}$~*N*(0, *Q*). The prediction Equation (1) will go along with an observation equation to describe the correlation between the measured value and the prediction at the discrete-time ***k***:

(2) $$Z_{k}=HX_{k}+V_{k}.$$

in which, $Z_{k}$ is the observation vector or measurement vector; *H* is the observation matrix and $V_{k}$ is the observation noise vector with the observation covariance matrix *R,* $V_{k}$~*N* (0, *R*). The *A*, *B*, *H*, *Q* and *R* can have the subscript index ***k*** if they change with discrete-time, yet they are invariant in most problems. Figure 1a illustrated the Kalman algorithm. In the diagram, $P_{k}^{+}$ and $P_{k}^{-}$ are the updated and predicted state error covariance matrices respectively [37]. $X_{k}^{-}$ is called an a priori prediction. The output from the Kalman is the updated $X_{k}^{+}$ or an a posteriori vector.

There are many systems that their state equation is nonlinear, so Equation (1) cannot be applied. The state can depend on a certain function ***f*,** which is:

(3) $$X_{k}^{-}=f\left(X_{k-1}^{+},\;U_{k},\;W_{k-1}\right).$$

The observation equation of the measurement and prediction vectors can be:

(4) $$Z_{k}=h\left(X_{k}^{-},V_{k}\right).$$

To be able to apply the Kalman filter, a linearization was suggested to approximate the nonlinear problem into a linear problem by first-order Taylor series. At each discrete-time, it is essential to calculate:

(5) $$F_{k-1}={\frac{\partial f}{\partial X}|}_{(X_{k-1}^{+},\;U_{k},\;0).}$$

(6) $$h_{k}={\frac{\partial f}{\partial X}|}_{(X_{k}^{-},\;\;0).}$$

The extended Kalman filter (EKF) algorithm is shown in Figure 1b. Viewing the two algorithms in Figure 1a,b, the differences are in the prediction equations. The update equations are the same in both algorithms [30,37].

### 2.2. Neural Network

In this work, as the neural network was applied to fix the error data caused by the BG noise, the theory of the neural network was briefly discussed here. An artificial neural network or neural network (NN) copying the work of biological neural systems [38,39,40] can react with certain inputs to provides outputs. An NN can have many layers, and the number of nodes in each layer is arbitrary. Looking at layer *l* with *K* nodes, one can have the output equation of this layer is:

(7) $$a_{j}^{\left(l\right)}=\sigma \left(b_{i}^{\left(l\right)}+\sum_{r=1}^{R}w_{ij}^{\left(l\right)}.a_{i}^{\left(l-1\right)}\right)=\sigma \left(z_{j}^{\left(l\right)}\right),$$

where σ is an activation function such as linear function, binary step, hyperbolic tangent, sigmoid function, tanh, rectified linear unit (ReLU), softplus functions or Leaky ReLU [40,41]; $z_{j}^{\left(l\right)}$ = $b_{j}^{\left(l\right)}+\sum_{j=1}^{R}w_{ij}^{\left(l\right)}.a_{i}^{\left(l-1\right)}$ is the output of the *j*^th^ neuron of the layer (*l−*1); $W^{\left(l\right)}$ = {$w_{11}^{\left(l\right)},w_{12}^{\left(l\right)},\ldots ,w_{ij}^{\left(l\right)},\ldots ,\;w_{KR}^{\left(l\right)}$} and ***B*** = {$b_{1}^{\left(l\right)},b_{2}^{\left(l\right)}$, …, $b_{i}^{\left(l\right)}$, …, $b_{K}^{\left(l\right)}$} are weights and biases of the layer *l* respectively.

To have desired outputs from an input vector, the NN must be trained to find the weights and biases of the NN. The training process is actually an optimal problem of finding the global minima of a cost function, which is often based on the mean square error (MSE) [38,42,43]. NN has applications in many areas such as signal processing [44], voice recognition [45], image processing [46] or navigation [47]. The back-propagation algorithm (BPA) is a fundamental algorithm in NN. In this algorithm, it needs a set of training data including input vector ***X*** of *N* elements {$x_{1},\;x_{2},\ldots ,\;x_{N}$} and output vector ***O*** of *M* elements {$o_{1},\;o_{2},\ldots ,\;o_{M}$}. The quadratic cost function of the stochastic gradient descent (SGD) is defined as:

(8) $$C\left(O,Y\right)=\frac{1}{2}\|O{|}_{k}-Y{|}_{k}{\|}^{2}=\frac{1}{2}\sum_{m=1}^{M}{\left[o_{m}-y_{m}^{\left(L\right)}\right]}^{2}.$$

From Equation (8), the BPA to update the weight matrix and the bias vector for a hidden layer *l* is:

$$\begin{array}{l} E^{\left(l\right)}={\left(W^{\left(l+1\right)}\right)}^{T}.E^{\left(l+1\right)}\;⊙D^{\left(l\right)}\\ \Delta W^{\left(l\right)}{|}_{k+1}=-\eta .[E^{\left(l\right)}{|}_{k}.\;{\left(A^{\left(l-1\right)}{|}_{k}\right)}^{T}].\\ \Delta B^{\left(l\right)}{|}_{k+1}=-\eta .E^{\left(l\right)}{|}_{k} \end{array}$$

In which $\Delta W^{\left(l\right)}{|}_{k+1}$ and $\Delta B^{\left(l\right)}{|}_{k+1}$ are the update matrices for weights and biases of the hidden layer *l* respectively; $A^{\left(l-1\right)}$ is the output vector of the layer *l*-1; ${\left[D^{\left(l\right)}\right]}_{K\times 1}$ = $\sigma^{\prime }{\left[Z^{\left(l\right)}\right]}_{K\times 1}$ is the activation derivative matrix with the argument is $Z^{\left(l\right)}$ matrix of $z_{i}^{\left(l\right)}\;\mathrm{and}\;E^{\left(l\right)}$ is the error matrix. η is the learning rate. If η is too small, it may take a long time to find the global minima. If η is large, it can never obtain the optimum global minima. To overcome this difficulty, the steepest descent algorithm was proposed by using Taylor approximation to find an appropriate η [39]. In this algorithm:

(9) $$E\left(W+\eta d\right)\approx \;E\left(W\right)+\eta .g^{T}.d,$$

where, g is the vector gradient of E(W), and **d** is the descent direction. η should be small enough to make E(W+ηd)−E(W)<0. Since η should not be so small, η can be chosen to minimize E(W+ηd). Thus, $E_{\eta }^{'}=0\to g^{T}.d=0\to [\begin{array}{c} g=0\\ g⊥d \end{array}$. To increase the convergence speed, Newton algorithm can be used. In this algorithm, the update form of the weights is:

(10) $$W^{\left(l\right)}{|}_{k+1}=W^{\left(l\right)}{|}_{k}-\eta_{k}.H^{-1\left(l\right)}{|}_{k}.\;{g|}_{k},$$

where, ${H^{\left(l\right)}|}_{k}=\nabla^{2}E\left(W^{\left(l\right)}{|}_{k}\right)$ is the Hessian matrix. Solving the equation of ${H^{\left(l\right)}|}_{k}.{d|}_{k}=-{g|}_{k}$ to find the descent direction ${d|}_{k}$ at discrete time *k*. Equation (9) is applied to find $\eta_{k}$ [39]. For the least square problems, as the Hessian matrix calculation is difficult sometimes, the Levenberg–Marquardt algorithm (LMA) can be applied to avoid that calculation by the approximation of $H=J^{T}.J$, in which, J is the Jacobian matrix of first derivative $\nabla E\left(W^{\left(l\right)}{|}_{k}\right)$ [39]. In our NN, the LMA was applied to find weights and biases.

## 3. Methodology

### 3.1. System

**Figure 2:**
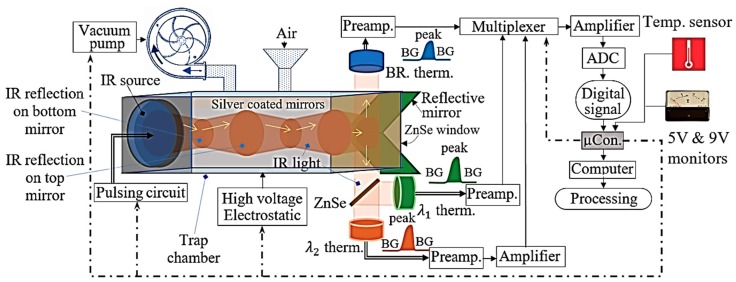
High voltage trap chamber and the thermopiles, circuit of the amplifiers and operation diagram.

The Fusarium detection device was upgraded from the authors’ previous work, which was presented in [20] by removing the reference chamber or the splitting plate to make only one reaction chamber. The trap has two silver-coated mirrors at the top and bottom, one IR source, one ZnSe window, a pair of reflective mirrors to direct IR light to the IR thermopiles, an inlet pipe, an outlet pipe and methyl methacrylate plates to cover the surrounding. The upgraded device structure is shown in Figure 2. The reference or broadband (BR) thermopile became the third detector along with the other two thermopiles to analyze the incident IR light. The broadband thermopile has the IR spectrum of 1 µm to 20 µm; $\lambda_{1}$ and $\lambda_{2}$ thermopiles have very narrow bandwidth spectra by using window filters of 6.09 ± 0.06 µm and 9.49 ± 0.44 µm respectively. The window filters were supplied by Northumbria Optical [48] and installed into the 2 mm × 2 mm 2 M thermopiles supplied by Dexter Research Inc. [49]. The typical internal resistance of these thermopiles is about 10 kΩ, and the responsivity R is 18.9 V/W. From [49], the damage threshold P_thres_ is 0.5 W/cm^2^, so it is not recommended to expose the 2 M thermopiles to any IR source higher P_thres_. The IR source is 2.2 mm × 2.2 mm JSIR350-4-AL-C-D3.7-2-A5-I, and its spectrum is around from 1 µm to 20 µm [50]. In the measurement, the biased current and the voltage for the IR source were 141.4 mA and 5.65 V respectively. As the signals from the thermopiles in this research were extremely weak, preamplifiers were necessary. The preamplifiers employed the AD8629 integrated circuit (IC) devices because these ICs have low bias current, low offset voltage, high common-mode rejection ration as well as chopping stabilization circuit [51]. These features will help to lower the output noises. The final amplifier is OPA320 IC [52]. The output of the final amplifier is digitalized by a 24-bit LT2400 analog-to-digital converter (ADC) [53]. The setup voltage for the ADC was 4.096 V, so the resolution was 0.488 µV [20]. In the device, a vacuum pump was attached to the inlet pipe. An output of a 15 kV high voltage (HV) circuit was connected to one of the silver-coated mirrors. In the device, to monitor the operating conditions of the device, a temperature sensor DS18B20 and a 5 V monitor and a 9 V monitor were used to monitor the output of the regulator circuits. When the temperature of the environment and voltages of these regulators change, the changes will be recorded to serve for the data error correction.

The microcontroller (µC) used to operate the system is an Atmelt 328p [54]. In Figure 2, to start, the µC turns on the vacuum pump to deliver the air into the trap chamber. The particles in the air are caught by the electrostatic charges provided by the HV circuit. After turning off the pump and the HV module, the microcontroller starts to collect data by following the following measurement procedure:

- Phase 1: Measuring environment temperature—T1; then, measuring outputs of the 5 V and 9 V regulators, which are V1 and V2 respectively.
- Phase 2: Measuring background data of BR thermopile in 6 s, when the IR source is still turned OFF; turning ON the IR source in 1.5 s and measuring data from the BR thermopile during this period to have peak data (PD); turning OFF the IR source in 6 s and measuring background data of the BR thermopile again. Thus, the data include background data, peak data PD and background data again.
- Phase 3: Similar to phase 2, $\lambda_{1}$ thermopile data are measured.
- Phase 4: Similar to phase 2, $\lambda_{2}$ thermopile data are measured.
- Phase 5: Repeating phase 1, but renaming temperature as T2, and the outputs of 5 V and 9 V regulators as V3 and V4 respectively.
- Phase 6: Sending all data to the computer in time order for further processing and analyzing.

In the computer, the background data will be averaged to have BG mean value. The data order is T1-V1-V2-BG-PD-BG-T2-V3-V4. After the measurement, one will have one data batch. To have a precise analysis, this procedure can be repeated to have more data batches. The number of the measurement batches is arbitrary. To have a good decision, five batches are sufficient [20].

### 3.2. Analyzing Method

To be able to detect a sample in the device, it is necessary to find a formula that depends only on the monochromatic absorbance features of the samples. From Beer–Lambert law, we proposed a group distinction coefficient equation, which can be applied to distinguish a group of samples in the device as follow [20]:

(11) $$\eta =\frac{\varepsilon_{\lambda_{1}}}{\varepsilon_{\lambda_{2}}}=\frac{log\left(\frac{P_{\lambda_{1}}}{P_{o,\lambda_{1}}}\right)}{log\left(\frac{P_{\lambda_{2}}}{P_{o,\lambda_{2}}}\right)}.$$

In which $P_{o,\lambda }$ is the IR radiant power of a monochromatic light of the IR light source (W/sr); $P_{\lambda }$ is IR power of the monochromatic light going through a sample (W/sr) and $\varepsilon_{\lambda }$ is monochromatic extinction coefficient (1/obj.). The formula to determine the density of the sample is [20]:

(12) $$D_{x}=\frac{log\left(\frac{P_{x,\;\lambda_{1}}}{P_{\mathrm{xo},\lambda_{1}}}\right)\;}{\varepsilon_{\lambda_{1}}*S}=D\times \frac{\log\left(\frac{P_{x,\lambda_{1}}}{P_{\mathrm{xo},\lambda_{1}}}\right)}{\log\left(\frac{P_{\lambda_{1}}}{P_{o,\lambda_{1}}}\right)},$$

where $D_{x}$ is an unknown density of a sample; D is a known-sample density (obj./cm^2^); S is the area of the sample and obj. is the studied object, which is caught on the area S.

Additionally, from experiment results, the Fusarium curve of P_BR_ and D_x_ can be plotted and in the later measurements, the values of P_BR_ and D_x_ can be found. Testing whether the data point of (P_BR_, D_x_) is on the curve can consolidate a decision of detection. This additional step helps to eliminate the confusion between two samples having a similar group-distinction coefficient η. Therefore, the third sensor is added to improve the reliability and extend application areas.

Power of incident light coming to a thermopile can be calculated by applying:

(13) $$P_{inc}=\frac{N_{dig}*resolution\left(µV\right)}{responsivity\left(\frac{V}{W}\right)*Gain},$$

where, $N_{dig}$ is digital output from ADC when reading thermopile data. Actually, when $\frac{P_{x,{\;\lambda }_{1}}}{P_{\mathrm{xo},\lambda_{1}}}$ ratio is estimated, the resolution, responsivity, and gain will cancel out each other. Therefore, $\frac{P_{x,\;\lambda_{1}}}{P_{\mathrm{xo},\lambda_{1}}}=\frac{N_{dig-x,\;\lambda_{1}}}{N_{dig-xo,\lambda_{1}}}$.

### 3.3. Adaptive and Cognitive Kalman Filter

In our work, the Kalman filter had two functions, which were the noise filter and outlier reducer for the signal data of each thermopile. As mentioned in Section 2.1, Q is the process noise covariance. In our work, the Kalman filter processed signal data of each thermopile, and Q should be called as the process noise error. The process noise, theoretically, depends on the working condition at each discrete-time, but in many problems, this condition is almost unchanged. During the time of turning ON the IR source, the IR radiation changed the working condition, so the process noise errors in the turning ON and turning OFF periods were not the same. The observation error could be determined from the experiments.

**Figure 3:**
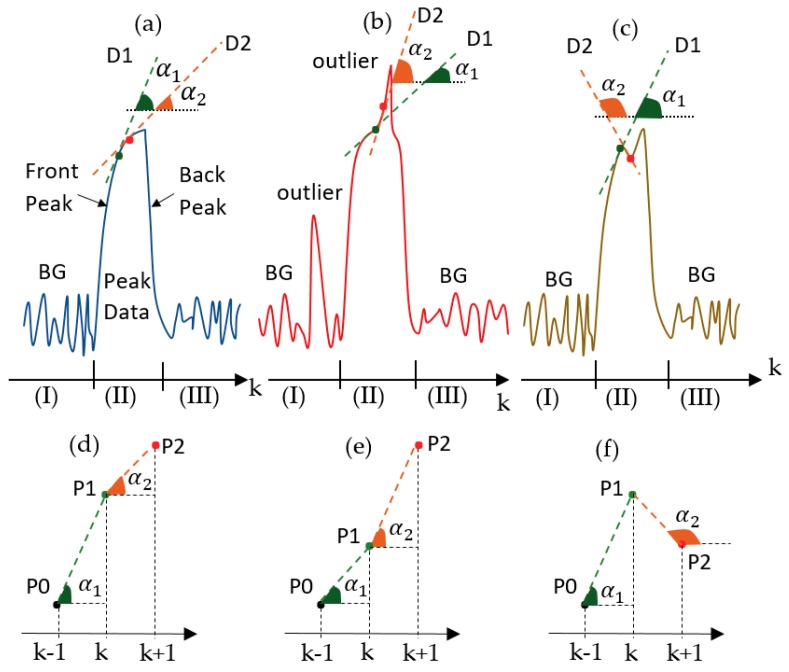
Three typical types of pulse data can be seen in the collected data. (**a**) Normal pulse data; (**b**) abnormal pulse data with positive outliers in the background and in the peak; (**c**) abnormal pulse data with a negative outlier in the peak and (**d**–**f**) close view of tangential line angles $\alpha_{1}$ and $\alpha_{2}$ of cases (**a**), (**b**) and (**c**) respectively.

Section 3.1 described the measurement procedure. In a turning ON period, the temperature of the IR source promptly increased. Since the IR source used the microelectromechanical system (MEMS), the temperature would soon reach the saturation temperature. As a result, in the early of the turning ON period, the signals on the thermopiles increased quickly but slightly improved in the end of this period. In the turning OFF period, the IR source temperature quickly decreased until reaching the environment temperature, so the data in this period would decline too. In practice, three types of data pulse can be seen as illustrated in Figure 3.

Figure 3a illustrates a normal pulse, in which, the front peak (FP) corresponds to turning OFF and the back peak (BP) corresponds to turning OFF and the START of the temperature balance period. In the research, the burst or popcorn noise may occur during the data collection and cause outliers in background and PD. Figure 3b,c shows the two typical pulse data with burst noise or outliers. As the front peak data caused by the reaction of the thermopiles with the coming-IR light reflecting from analyzing samples, the data will contain useful information of the samples. Besides, from observation, outliers often appear in FP range. Therefore, we focused on how to process outliers in the FP range. In the FP range (illustrated in Figure 3a), let us look at two adjacent points, P1 and P2 corresponding to the discrete-time k and k + 1, in a data peak. D1 and D2 are the tangential lines going through P1 and P2 respectively. $\alpha_{1}$ and $\alpha_{2}$ are the angles of the tangential lines D1 and D2 with the horizontal line. For normal pulses, it can be seen that:

(14) $$\{\begin{array}{c} 0\leq \alpha_{1},\alpha_{2}<90^{o}\\ \alpha_{2}<\alpha_{1}\to tan\left(\alpha_{2}\right)<tan\left(\alpha_{1}\right) \end{array}\to \frac{P2-P1}{\Delta t}>\frac{P1-P0}{\Delta t},$$

where:

(15) $$tan\left(\alpha_{1}\right)=\frac{P1-P0}{t_{k}-t_{k-1}}=\frac{P1-P0}{\Delta t};\;tan\left(\alpha_{2}\right)=\frac{P2-P1}{t_{k+1}-t_{k}}=\frac{P2-P1}{\Delta t}.$$

Similarly, the conditions for Figure 3b,e are:

(16) $$\{\begin{array}{c} 0\leq \alpha_{1},\alpha_{2}<90^{o}\\ \alpha_{2}>\alpha_{1}\to tan\left(\alpha_{2}\right)>tan\left(\alpha_{1}\right) \end{array}\to \frac{P2-P1}{\Delta t}>\frac{P1-P0}{\Delta t}.$$

For Figure 3c,f, the conditions are:

(17) $$\{\begin{array}{c} 0\leq \alpha_{1}<90^{o},90^{o}<\alpha_{2}\leq 180^{o}\\ \alpha_{2}>\alpha_{1}\to tan\left(\alpha_{1}\right)>0\;\&\;tan\left(\alpha_{2}\right)<0 \end{array}\to \frac{P1-P0}{\Delta t}>0\;\mathrm{and}\;\frac{P2-P1}{\Delta t}<0.$$

The conditions in Equations (14), (16) and (17) can be used to determine normal or abnormal pulses in the FP range. P0, P1 and P2 are the digital values of FP range. (P1–P0) and (P2–P1) could be calculated by applying the firs-order discrete derivative of the pulse, and [(P2–P1)–(P1–P0)] is the second-order discrete derivative of the pulse. Let us name ***f*** as the function of the peak, so the first and the second-order derivative by discrete-time k are $\dot{f}$ and $\ddot{f}$ respectively. Then (P1–P0) and (P2–P1) become $\dot{f}$(k−1) and $\dot{f}$(k), respectively; [(P2–P1)–(P1–P0)] = $\ddot{f}$(k).

**Figure 4:**
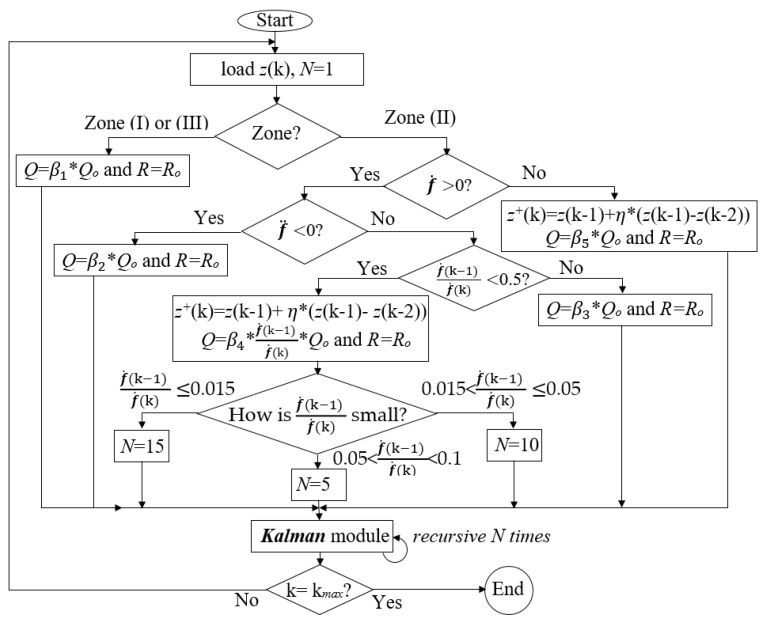
The algorithm of the adaptive-cognitive Kalman filter (ACKF). Based on *N*; the Kalman can recall itself *N* times.

As mentioned above, in the turning ON and OFF periods, the process noise and the other parameters of the Kalman filter should be adjusted. Figure 4 shows the adjustment diagram of process noise, and recursive coefficients based on the experiments, the conditions in Equations (14), (16) and (17) for the ACKF.

Let us name *Q_o_* and *R_o_* as the constant process noise and observation noise errors respectively. In the discrete-time zones (I) and (III), the data are BG data. In these discrete-time zones, the process noise error is set at *Q* = $\beta_{1}$ × *Q_o_* and the observation noise error is *R = R_o_*. Attentionally, *R = R_o_* everywhere, and the values $\beta_{1}$, $\beta_{2}$, $\beta_{3}$, $\beta_{4}$ and $\beta_{5},$ which will be discussed later are cognitively determined by experiments. Figure 3b shows an example of background range with an outlier that can be fixed by the Kalman filter if $\beta_{1}$ is appropriately chosen. In the discrete-time (II), the FP range is studied. In the FP range, if $\dot{f}$(k) > 0 condition is true, the condition in Equation (16) is considered:

(18) $$\to \;\frac{P2-P1}{\Delta t}-\frac{P1-P0}{\Delta t}>0\to \;\left(P2–P1\right)–\left(P1–P0\right)>\;0\to \ddot{f}\left(k\right)>0.$$

If $\ddot{f}\left(k\right)<0$, it is normal and *Q* = $\beta_{2}$ × *Q_o_*. If $\ddot{f}\left(k\right)>0$, it is abnormal and an outlier appears in the FP range. If the outlier is large, it requires a correction for the observation value. In our research, if $\frac{\dot{f}\left(k-1\right)\;}{\dot{f}\left(k\right)}\geq$ 0.5, then the outlier is not large → *Q* = $\beta_{3}$ × *Q_o_*. If $\frac{\dot{f}\left(k-1\right)}{\dot{f}\left(k\right)}<$ 0.5, then the outlier is large. The observation correction is conducted by using the previous normal data points at discrete times k − 1 and k − 2:

(19) $$z^{+}(k)=z(k-1)+\eta \times (z(k-1)–(k-2)),$$

where *z^+^*(*k*) is the observation prediction and η is a percentage constant to take an amount of the difference of *z*(*k*-1)–*z*(*k*-2). After this prediction, we still put more reliability on the process noise error rather than the observation noise error. In other words, at discrete time k, the observation noise error should be larger than the process noise error (*R* > *Q* or $\frac{Q}{R}<1$; *R = R_o_*). It can be seen that:

$$\frac{P1-P0}{P2-P1}<1\to \frac{Q}{R_{o}}\sim \frac{P1-P0}{P2-P1}\to Q\sim \frac{P1-P0}{P2-P1}=\frac{\dot{f}\left(k-1\right)\;}{\dot{f}\left(k\right)}.$$

Thus, *Q* = $\beta_{4}$ × $\frac{\dot{f}\left(k-1\right)\;}{\dot{f}\left(k\right)}$ × *Q_o_*, and *Q* can adapt to the magnitude of $\frac{\dot{f}\left(k-1\right)\;}{\dot{f}\left(k\right)}$. In addition, a recursive mechanism is designed to recall the Kalman filter module itself. The number of recalls, *N*, depends on whether this ratio is small or large. The smaller $\frac{\dot{f}\left(k-1\right)\;}{\dot{f}\left(k\right)}$ is, the more the Kalman module will recall itself. Basically, even in a normal case, the Kalman filter is called two times, so *N* = 2. If 0.05 < $\frac{\dot{f}\left(k-1\right)\;}{\dot{f}\left(k\right)}$ < 0.1, *N* = 5. If $0.015<\frac{\dot{f}\left(k-1\right)\;}{\dot{f}\left(k\right)}\leq$ 0.05, *N* = 5. If $\frac{\dot{f}\left(k-1\right)\;}{\dot{f}\left(k\right)}\leq 0.015$, *N* = 15. If $\dot{f}$(k) < 0, a negative outlier occurs in this range. The observation data point is abnormal, and it will be corrected by applying again Equation (19). The process noise error is *Q* = $\beta_{5}$ × *Q_o_*. After being processed, the outlier point becomes a normal data point. If the outlier still exists in the FP range, it will be detected and processed. After being processed by the ACKF, thermopile data are symbolized as [BG, PD, BG]_preprocessed_.

### 3.4. Entropy

To evaluate the effectiveness of the filter and outlier-elimination process, the entropies of the raw and preprocessed signals is used:

(20) $$S(y)=\sum_{i}p\left(i\right)*\log_{2}\left(\frac{1}{p\left(i\right)}\right).$$

In which *y* can be *x*, the raw data, or *z*, the processed signal data, and p(i) is the probability of *x*(*i*) or *z*(*i*) to happen [55,56,57]. Entropy quantity can reveal the uncertainty or the randomness of the investigated signal. To the raw signal containing much noise, the noise can cover the useful information and show a high disorder, so the entropy of the signal is small. If the outlier elimination modules work well, much noise including thermal or burst noise is reduced, then the entropy of the preprocessed signal can be larger than the raw signal.

In the NN training, as the BG noise of each thermopile affects most to the output, it is crucial to choose a standard BG (SBG), and corresponding with each SBG is a standard MP (SMP) based on the measurements of each thermopile. The SBG for each thermopile is chosen based on the appearance frequency of the BG data. The chosen BG should be the highest appearance frequency. We use the absolute-mean error function (AME as a stop criterion and efficiency coefficient). AME equation is:

(21) $$\mathrm{Error}\;=\;mean\left(\sum_{i}\mathrm{abs}\left(\mathrm{SMP}-y\left(i\right)\right)\right).$$

However, we encountered some cases that the correction values swing around the SMP. To overcome the problem, applying Equation (20) of the entropy provides a better operational condition. As discussed above, to a data with much noise or a fluctuation data vector, the entropy will be small. In the training, the program will train NN and drive corrected data to a trend of entropy increase. Therefore, the best entropy will lead to the least swinging correction values.

### 3.5. Error Correction by Neural Network

In Section 3.1, temperature, 5 V and 9 V monitoring voltages and raw data were presented. Section 3.3 introduced the data after being preprocessed. Although the thermal and burst noises can be mitigated by the ACKF filter, the BG noise or error still exists in the data. To reduce this noise, a NN was applied. The NN was trained by a set of collected data from the *Fusarium* detection device. To prepare data for training NN, some estimations should be done first: $\bar{T1}=\frac{T1+T2}{2}$, $\bar{V1}=\frac{V1+V3}{2}$ and $\bar{V2}=\frac{V2+V4}{2}$; BG is the average of background data; STD(BG) is the standard deviation of background data; MP is the maximum of PD; WP is the mean value of the whole PD and FP is the mean value of data points in FP range. To train the NN precisely, many data batches were recorded. Each data batch will have the previously introduced quantities. Gathering data for these quantities from the measured data batches, one will have data vectors, which are presented in bold font: $\bar{T1}$, $\bar{V1}$, $\bar{V2}$, **BG**, **STD**(BG), **MP**, **FP** and **WP**. **MP** vector is used to analyze samples in the trap chamber (Section 3.1), and is the *N*_dig_ data in equation (13) (Section 3.2).

Theoretically, if the operating conditions and the studied sample are unchanged, **MP** will be stable. However, the operating condition set (**OCS**) of $\bar{T1}$, $\bar{V1}$, $\bar{V2}$, **BG**, **STD**(BG), **FP** and **WP** are hardly stable, so **MP** are changed too. These quantities can affect to the **MP**. In the work, a standard operating condition set (SOCS) from **OCS** was chosen. Corresponding this SOCS is the three standard MP (SMP) values for the three thermopiles. If r = $\frac{\mathrm{SMP}}{\mathrm{MP}}$ is defined, then r depends on the OCS and SOCS. If *N* data batches are measured, then **MP** = {MP_1_, MP_2_, …, MP_i,_ …, MP_N_}. From **MP** vector, **r** = $\frac{\mathrm{SMP}}{MP}$ vector can be calculated. From Section 3.1, we know that MP is the digital value of amplified signal from an input signal X. MP can be estimated by: MP = *G.*X, where, *G* is the gain of the amplifiers. As an OCS can affect to the gain, so *G* depends on the OCS. Therefore, SMP = *G_S_*.X, where *G_S_* is the gain at standard condition set. If X is stable, the ratio of MP_i_ is:

(22) $$r_{i}\;=\;\frac{\mathrm{SMP}}{{\mathrm{MP}}_{i}}=\frac{G_{s}}{G_{i}}.$$

**Figure 5:**
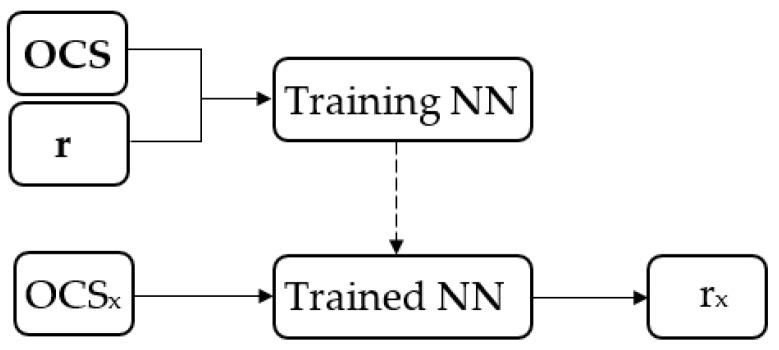
Training neural network (NN) and finding the ratio r_x_ diagram.

$G_{i}$ depends on the OCS at the measurement i^th^. The NN would be trained by using **OCS** of $\bar{T1}$, $\bar{V1}$, $\bar{V2}$, **BG**, **STD**(BG), **FP**, **WP** and **r**. The trained NN would be used to determine r_x_ from a new OCS_x_ of a new investigation of any new sample. These procedures are depicted in Figure 5.

From r_x_, MP_x_ of the new sample can be corrected to eliminate the affection of OCS_x_ by applying Equation (22), so MP_x_ is adjusted to SMP, which is the MP value corresponding the SOCS:

MP_corrected_ = SMP = r_x_ × MP_x._ (23)

**Figure 6:**
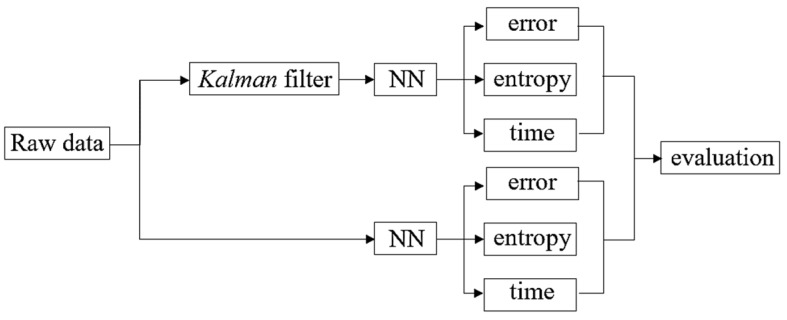
Estimation of the effectiveness of the ACKF.

In NN training, the input data and output data to supply into the NN were recorded in two cases of without-sample and *Fusarium* sample and in different operating conditions. In this paper, we mainly focused on the operation of the ACKF and the role of the broadband thermopile in the upgraded nondispersive thermopile device. The collected data would be preprocessed by the ACKF, and then being used to train the NN. To evaluate the effectiveness of the ACKF filter, the NN would be trained by two **OCS**s of raw data and ACKF-preprocessed data. Based on the comparison of the errors, entropies and times of NN training after employing the two **OCS**, the effectiveness can be concluded. The diagram of using the collected data for NN training is shown in Figure 6.

### 3.6. Samples

The samples were used in the experiments are *Fusarium oxysporum* [22] and starch as these two samples have the group distinction coefficients are close to each other. In the previous work, we also used pollen and turmeric to test the device and the analyzing formula, which is the group-distinction coefficient [20]. The *F. oxysporum* was collected from rotten garlic bulbs and nurtured in potato-dextrose-agar Petri dishes by following the instructions in [22]. To be able to collect *Fusarium* samples, it requires at least 4 weeks of fostering. The starch sample was from a local food market. The samples were used to test if the outlier reduction by ACKF and the upgraded *Fusarium* detection device can work effectively.

## 4. Results and Discussion

In [20], four samples were used to test the *Fusarium* detection method and device. In that research, the coefficients of *Fusarium*, pollen, starch and turmeric were 1.144 ± 0.153, 0.136 ±0.116, 0.939 ± 0.073 and 0.794 ±0.139 respectively. It can be seen that *Fusarium* and starch coefficients were very similar to each other. Therefore if there is a way to process further the samples with similar coefficients, it will be useful. In this work, we focused on mainly the method of using the combination of BR, $\lambda_{1}$ and $\lambda_{2}$ thermopiles to distinguish the two samples, *Fusarium* and starch, which have similar group-distinction coefficients.

### 4.1. Reduction of Thermal and Burst Noises

**Figure 7:**
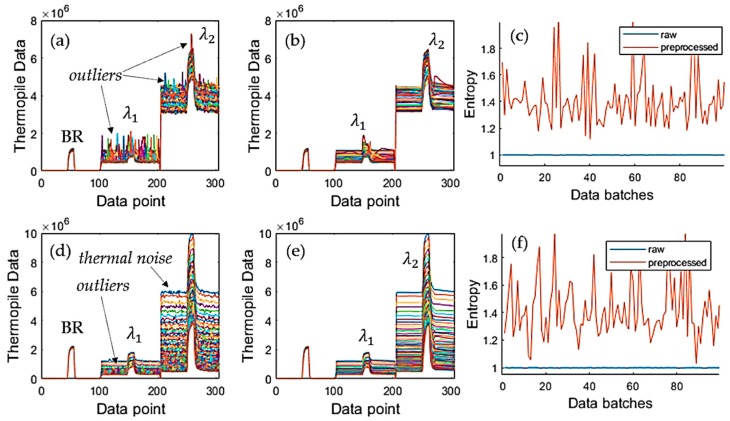
One hundred raw signals and their ACKF preprocessed signals when applying the ACKF in two different measurement sets. (**a**,**d**) Raw signal; (**b**,**e**) preprocessed signal and (**c**,**f**) entropies of the first-order differentiate corresponding to each signal.

This section presents the operation of the ACKF. Its results and the raw data are shown in Figure 7. Figure 7a,d shows thermopile signals with noise and outliers. Especially, Figure 7a has many outliers. Figure 7b,e shows the preprocessed signals by applying ACKF to filter out the noise and the outliers. Figure 7c,f depicts the entropies of the first-order differentiation of these signals. Each entropy value will stand for an uncertainty level of a signal. As seen, the ACKF work well, a few outliers still can be seen in Figure 7b.

**Figure 8:**
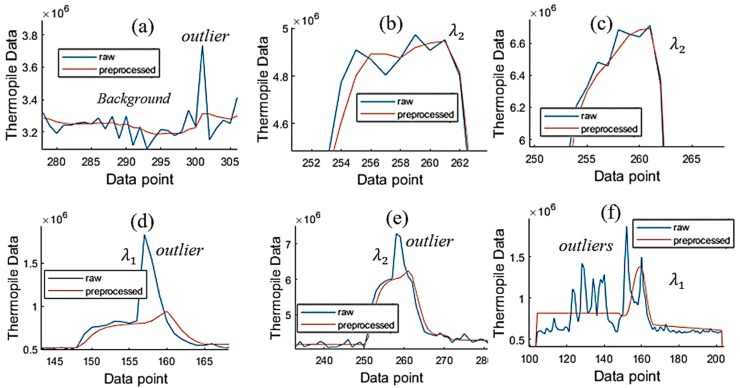
Close views of background, $\lambda_{1}$, and $\lambda_{2}$ of the raw and preprocessed signals. (**a**) Background; (**b**,**c**,**e**) $\lambda_{2}$ thermopile signals and (**d**, **f**) $\lambda_{1}$ thermopile signals.

Figure 8 illustrates some cases showing a better view of the effectiveness of the ACKF. The outliers can happen in the background or peak zones as shown in Figure 8a,d–f. In these plots, Figure 8f could not be fixed well as the signal had too much affection from the thermal and burst noises. Figure 8b,c did not get much effect from the burst noise and the ACKF function was to smooth the raw signals.

Table 1 shows the max peak (MP) differences, ΔMP, between the MP of ACKF the preprocessed and raw signals of the three thermopiles. Similarly, it also introduces the entropies of the signals of a typical case of the outlier effect. It can be seen that $\lambda_{2}$ raw signal had an outlier in the peak. Thus, ΔMP of $\lambda_{2}$ was very large, while ΔMPs of BR and $\lambda_{2}$ were very small. The last two columns show the entropies of the signal differentiation of the three thermopiles of the raw and preprocessed signals. Table 1 proves that entropies of differentiation of the ACKF preprocessed data were better than the raw data. Thus, the ACKF could process the thermal noise and burst noise well.

### 4.2. Reduction of Background Noise

For training data of the NN, 5422 data were consecutively and automatically recorded in many days to mimic normal working conditions. To find an appropriate and adequate NN structure for our application, we simply started using a single hidden layer with two nodes, and then the number of nodes was increased. The number of nodes was stopped at eight. The training times and absolute errors from the training were taken note. Then, we increased the number of hidden layers to two layers with m = 2 nodes for the first hidden layers and n = 1 node for the second layers; m was increased until reaching eight nodes for the training. Then, n was increased to two nodes, and again m started at m = 2 nodes. After m = 8 nodes and n = 2 nodes, we stopped there and compared the times and errors in the simulation to find the best NN structure.

The best NN structure had two hidden layers, in which, the first hidden layer had three nodes, while the second had two nodes. To compare the effectiveness of the ACKF, the raw data and the preprocessed data were employed. The data aggregation was of five different samples in which there were no sample, *Fusarium* samples at different densities and starch sample. In each case, the power supply for the IR source and the other circuits were unchanged, so the outputs of the three thermopiles were expected constant. Additionally, the number of batches in each sample-measurement case was arbitrary. However, the working condition was probably unstable and even the power supply could have a certain fluctuation, which could affect the output of the detectors. By using the inputs of the information of the temperature, 5 V and 9 V monitors and the BG to train the NN, we could correct the recorded-unstable outputs of these thermopiles, and return back more stable outputs. Firstly, we checked the efficiency of the ACKF by comparing the training times and the absolute errors of the $\lambda_{1}$ and $\lambda_{2}$ thermopiles. The results are depicted in Table 2.

**Figure 9:**
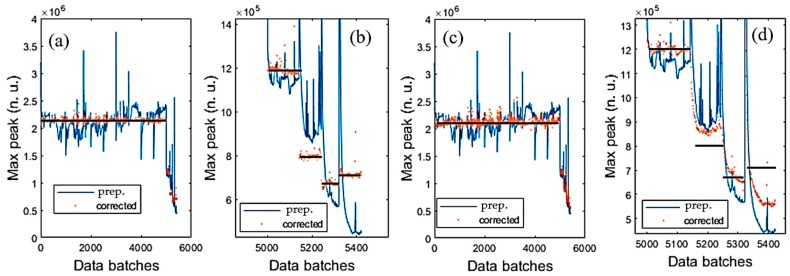
The ACKF preprocessed (prep.) and corrected max peak (MP) data of $\lambda_{1}$ thermopile of using entropy and absolute-mean error function (AME) criteria respectively. (**a**) Full view of the data achieved by entropy criterion; (**b**) close view of the data batches from 5001 to 5422 achieved by entropy criterion; (**c**) full view of the data achieved by AME and (**d**) close view of the MP data from the batches of 5001 to 5422 achieved by AME criterion.

In the training NN, both error and entropy criteria were applied. As mentioned in the entropy section that the AME may cause the data correction swing even though the AME was optimized through the weights and biases searching. Figure 9 shows the plots of the ACKF preprocessed data and corrected data of $\lambda_{1}$ thermopile of using error and entropy as operational criteria.

In the preprocessed MP data of the $\lambda_{1}$ thermopile, Figure 8a illustrates the results of applying entropy. Figure 9b is the close view of Figure 9a of the four different samples. Similarly, Figure 9c,d show the results when using the AME criterion. The black lines in the plots are to show the expectation of MP values. The expectation MPs were chosen from view the correlation of the MP data and the SBGs of the three cooperative thermopiles. The close views show data of the other four different samples. It can be seen the entropy operating criterion could work better than the error operating criterion.

**Figure 10:**
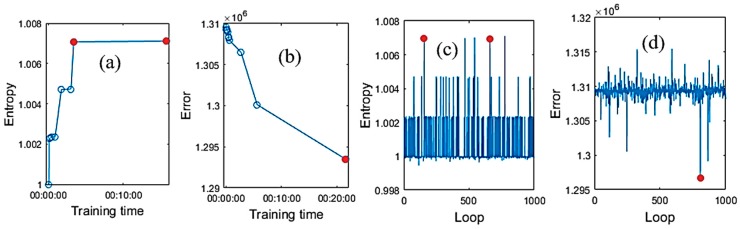
The entropies and AMEs were achieved from the training NN, which was trained in 1000 loops for $\lambda_{1}$ thermopile. The red dots show the optimization values. (**a**) Entropies from applying entropy for the differentiated MP data; (**b**) errors from applying the AME criterion; (**c**) recorded entropies after 1000 loops and (**d**) recorded errors after 1000 loops.

Figure 10 shows the other views on the operation of these criteria when processing the $\lambda_{1}$ thermopile MP data. Figure 10a,b presents the relationship between the training time and entropy of differentiation, and error of the corrected data respectively. The two red dots in Figure 10a,c are the two optimized entropies, which are close to each other. Figure 10c,d are the results that were recorded in one searching batch of 1000 loops.

**Figure 11:**
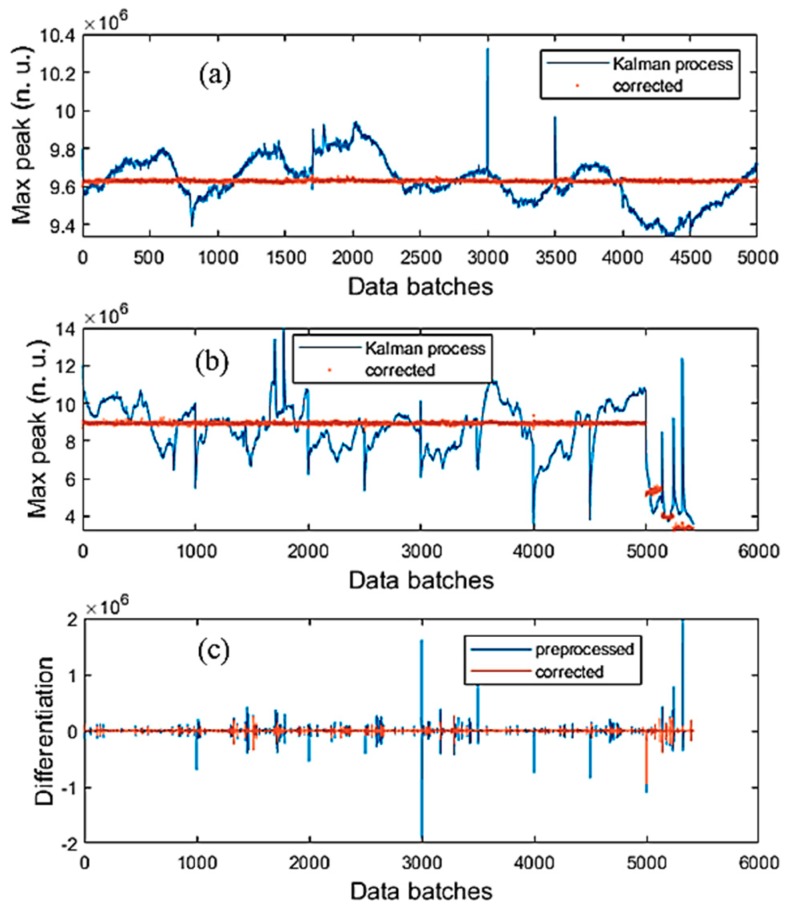
The ACKF processed data, the corrected data, and the differentiation of these types of data. (**a**) Broadband (BR) thermopile; (**b**) $\lambda_{2}$ thermopiles and (**c**) differentiation of the preprocessed and corrected data of $\lambda_{1}$ thermopile.

Figure 11 shows the results of processing BR and $\lambda_{2}$ thermopiles, and the differentiation plots of the data. Figure 11a is of the BR thermopile and Figure 11b is of the $\lambda_{2}$ thermopile. A note that the entropy was applied to the differentiation of the preprocessed data and the corrected data. The differentiation plots of the two types of data shown in Figure 11 belong to the $\lambda_{1}$ thermopile. Table 3 shows the results of applying entropy and AME operating criteria for $\lambda_{1}$ thermopile. In each method, both AME and entropy quantities were recorded for investigation. From Table 3, in the entropy operating criterion, when the entropy was optimized, then the error was very close to the optimal error of the error operating criterion. However, in the error operating criterion method, it was not similar to the entropy, in this case, it was less than 1, which is not good. These points of view could be more consolidated by reviewing Figure 9 and Figure 10.

### 4.3. Analysis

**Figure 12:**
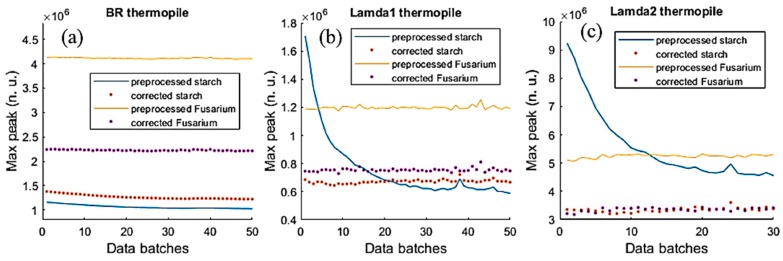
The ACKF preprocessed and corrected data of *Fusarium* and starch. (**a**) BR thermopile case; (**b**)$\;\lambda_{1}$ thermopile case and (**c**)$\;\lambda_{2}$ thermopile case.

From experiments, as the group distinction coefficients, η, of the *Fusarium oxysporum* chlamydospore [22], and the starch samples were somewhat similar, in this section, the analysis results of these samples were introduced. Applying the trained NN for these two samples can help to correct or calibrate the data of the three thermopiles. Figure 12 shows the ACKF preprocessed data and corrected data of the two samples, which were measured in 50 batches.

**Figure 13:**
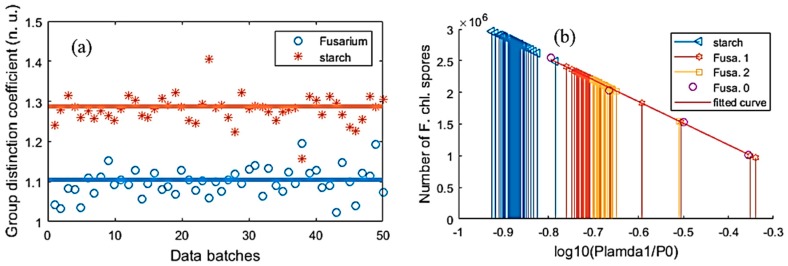
Using data of different *Fusarium* samples and starch sample measured by $\lambda_{1}$ thermopile. (**a**) $\eta_{starch\;}$ and $\eta_{Fusarium}$ and (**b**) the fitted curve of the known-in-advance *Fusarium* samples, the interpolation and extrapolation of the unknown-different *Fusarium* and starch samples. * Fusa. 0 stands for the known-in-advance *Fusarium* sample. Fusa. 1 and Fusa. 2 are two unknown-quantity samples.

From the figure, one can see that the output data of BR were very stable and the correction process calibrates the data. Applying Equation (11), the group distinction coefficients of the two samples could be found. Figure 13a shows $\eta_{starch}$ and $\eta_{Fusarium}$ plots. It can be seen that $\eta_{starch}$ and $\eta_{Fusarium}$ were very close to each other. To determine the number of Fusarium in the trap, Equation (12) was employed. Figure 13b depicts the relation of the number of *Fusarium* and *log*($\frac{P_{\lambda_{1}}}{P_{0,\lambda_{1}}}$) in case of applying data of $\lambda_{1}$ thermopile.

The fitted curve in Figure 13b was formed by applying data of the *Fusarium* samples, which their known quantities (N):

(24) $$f_{\lambda_{1}}=\mathrm{fitting}(\log(\frac{P_{\lambda_{1}}}{P_{0,\lambda_{1}}}),\;N).$$

As the group distinction coefficients of *Fusarium* and starch are close to each other, thus it can cause confusion at certain times. Table 4 shows the means of the group distinction coefficients, the absolute errors, and the relative errors of *Fusarium* and starch. From the table, one can see these values were very close to each other.

To improve the fidelity, the broadband thermopile was used. We investigated the other samples of starch and *Fusarium* that their quantities were unknown in advance. Making an assumption that all the samples were *Fusarium*, we could find the sample quantities N_x_ by replacing *log*($\frac{P_{\lambda_{1}}}{P_{\lambda_{1}0}}$) into $f_{\lambda_{1}}$ of Equation (24). Figure 13b illustrates the extrapolated and interpolated values of the new samples. From the data of the BR thermopile and the numbers of known-in-advance F. samples (Fusa. 0), the fitted curve was formed:

(25) $$f_{BR}=\mathrm{fitting}(N,\;\log(\frac{P_{\mathrm{BR}}}{P_{0,\mathrm{BR}}})).$$

Additionally, it is necessary to form the lateral fitted curves for the max and min data points, which can be seen from the error boxes. error1 and error2 are the errors of the numbers of the *Fusarium oxysporum* chlamydospore and log10($\frac{P_{BR}}{P_{0,BR}}$) respectively. Thus, the lateral-fitted curves are:

(26) $$f_{BR}max=\mathrm{fitting}(N+\mathrm{error}1,\;\log(\frac{P_{\mathrm{BR}}}{P_{0,\mathrm{BR}}})+\mathrm{error}2).$$

(27) $$f_{BR}min=\mathrm{fitting}(N–\mathrm{error}1,\;\log(\frac{P_{\mathrm{BR}}}{P_{0,\mathrm{BR}}})–\mathrm{error}2).$$

**Figure 14:**
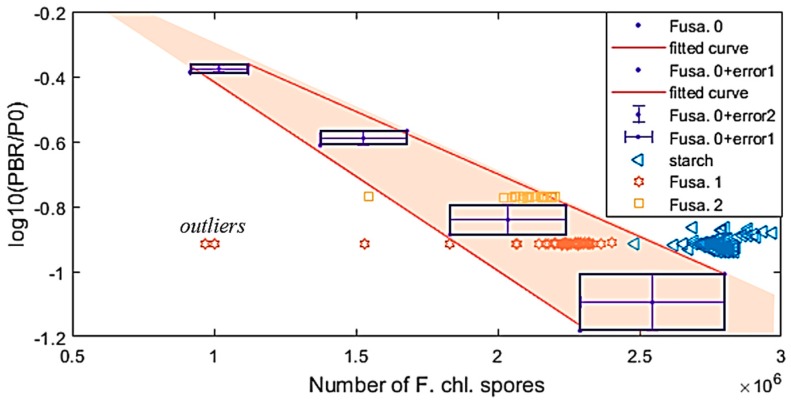
$f_{BR}\;$= fitting (N, *log*($\frac{P_{\mathrm{BR}}}{P_{0,\mathrm{BR}}}$)) and the validation area formed by the lateral curves of Equations (26) and (27).

In Equations (26) and (27), error1 and error2 are the errors of the quantity number of *Fusarium* sample N and *log*($\frac{P_{\mathrm{BR}}}{P_{0,\mathrm{BR}}}$) respectively. The lateral curves will create a validation area (VA). In the case of investigating new measurement, if the point of the quantity number N and *log*($\frac{P_{\mathrm{BR}}}{P_{0,\mathrm{BR}}}$) is in the VA and η of the sample is in the range of 1.125 ± 0.110, we could conclude that the sample is *Fusarium*. Drawing the points of (N_x_, *log*($\frac{P_{\mathrm{BR},x}}{P_{0,\mathrm{BR}}}$) ) is presented in Figure 14. The figure also provides a visual view of the lateral curve and the VA. In Figure 14, the points of starch samples were out of the VA, so along with η of starch, we could go to a conclusion with more confident and reliable. For the other two *Fusarium* samples, we could see that almost all of the measurement points were in the VA, except few points on the left of the figure.

### 4.4. Discussion

The ACKF helps to reduce thermal noise and burst noise well. To be able to fix the outliers of BG or peak data, at least some reference data points were not affected by the outliers. From these reference data points, the ACKF could eliminate the outliers. In reality, there are cases that the ACKF cannot fix the error data (Figure 8f), as the outliers happen too close to each other. Therefore, the reference data points are covered by the burst noise. As a result, the error data cannot adequately be fixed. Besides, from our experiments, we found that the ACKF could also help to reduce the time to search the global minima for the NN. This could be explained as the thermal noise and burst noise occurring in the BG noise were filtered very well by the ACKF (Section 4.1), so the NN could go to the global minima faster. The evidence of this point of view can be seen in Table 2.

Entropy is not only a useful tool to evaluate the work of Kalman filter, but also can be applied as an operational criterion to replace the other criteria such as the mean absolute error. The results in Section 4.1 and Section 4.2 show the efficiency of the entropy. From Figure 8c,d, and Table 2, although the error was the smallest after 1000 loops, the visual results were not what we expected. The corrected points fluctuated surrounding the expected lines. Entropy was applied to the differentiation of the preprocessed and corrected MP data rather than being applied directly to these data. Loosely speaking, the differentiation step helped to remove the difference in the magnitude of these MP data, as we only focused on the BG noise. The information left was mainly the BG noise, which influenced the MP data (Figure 10c). Entropy now reveals how much BG noise is removed by comparing the entropies of the differentiation of the preprocessed and corrected MP data.

Figure 12 presents the results when the trained NN was used to correct the error data of *Fusarium* and starch in which these samples were measured in many batches. In Figure 11a, one can see that the NN adjusted the MP of both samples. In Figure 11b,c, the MP data of starch achieved the largest errors as they lasted from around 0.6 × 10^6^ to 1.7 × 10^6^ in the case of $\lambda_{1}$ thermopile, and from around 4.3 × 10^6^ to 9.2 × 10^6^ in the case of $\lambda_{2}$ thermopile.

As the group-distinction coefficient of *Fusarium* and starch were very similar, the addition of another thermopile detector, the BR thermopile, could help to distinguish better these two samples. Figure 13 shows that some *Fusarium* points were out of the VA. This could be explained that in the data there were outliers, which the ACKF could not correct them. The figure also introduced a case that the starch point was in the VA. However, in general, one could see that most of the experiment points were in the VA, so the device could distinguish the *Fusarium* sample from other samples. With an adding detector, the ability of the device could be expanded. It could help to detect the group of many more substances.

The group-distinction coefficient of the starch was found in this work was a little bit different from the value in [20], 0.9390 ± 0.0732. This could be explained that the moistures of the starch samples used in this work and in [20] were different. A slight change in moisture of the starch sample might affect its group-distinction coefficient.

## 5. Conclusions

The proposed adaptive-cognitive Kalman filter worked well to reduce the thermal noise and burst noise. The background noise could be mitigated by applying a neural network. The entropy could be applied to replace the mean absolute error as an operational condition. The upgraded device increased the reliability and precision of the current *Fusarium* detection and quantifying by applying the proposed techniques. Additionally, by adding one more thermopile, the group coefficients of substances were more distinct. This assisted the device to distinguish different substances easier with higher accuracy compared to the use of only two thermopiles.

## Figures and Tables

**Table 1:** The investigation of the raw and preprocessed signals.

Illustration	Thermopile	ΔMP	Entropies of Diff. of Raw Signal	Entropies of Diff. of Preprocessed Signal
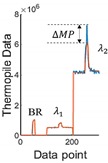	BR	1,162,693	0.9964	1.2808
	$\lambda_{1}$	9471	0.9993	0.9964
	$\lambda_{2}$	1873	0.9964	2.2958

**Table 2:** The training results of raw data vs. preprocessed (prep.) data.

	$\lambda_{1}$		$\lambda_{2}$	
	Raw data	Prep. Data	Raw Data	Prep. Data
Time	12 min 09 s	00 min 46 s	9 min 13 s	1 min 00 s
Error	2.7453 × 10^4^	1.4374 × 10^4^	2.3999 × 10^5^	1.76485 × 10^5^

**Table 3:** The operating coefficients of the entropy and error operating criteria.

	Entropy Operating Criterion	Error Operating Criterion
Time	15 min 47 s	21 min 37 s
Optimal entropy	1.0071	N/A
Optimal Error	N/A	1,293,496.24
Entropy	N/A	0.9999
Error	1.2935 × 10^6^	N/A

**Table 4:** Group distinction coefficient.

	Fusarium	Starch
η	1.125	1.31
Δη	0.110	0.06
$\varepsilon_{\eta }$	9.8%	4.6%
